# Diameter of the Red Blood Corpuscles

**Published:** 1877-11

**Authors:** 


### DIAMETER OF THE RED BLOOD-CORPUSCLES.

M. L. Perier, in a recent communication to the French Academy of Sciences, gives the results of his examination of the red blood-corpuscles for medico-legal purposes. His observations were made upon the blood of subjects whose ages ranged from the first moments of extra-uterine life to four score years. He says that

1. The diameter of the red globules can vary from .0031 to .0103 of a milimetre from birth to old age, and that it can slightly exceed the last figure in some individuals.

2. The extreme types of .0103 for the large corpuscles, (the giant globules of Hayem), and of .0031 to .0037 for the smallest are exceedingly rare. Those of .0103 were found but once and in a young girl of 19½ years; two infants just born and a little girl of eight months furnished the second type.

3. Globules of .0100 of a millimetre, which are found in great numbers in the first days of extra-uterine life, quickly disappear and are rarely afterwards seen. Those of .0045 are principally found in children and the aged.

4. The great mass of the globules, laying aside all considerations of age, temperament, and condition of life, measure between ,0050 and .0087 of a millimetre, or within the limits already established by former authorities.

5. The most numerous type, for its globules are always present, is represented by those of .0075. Those of .0087 come next in order of frequency. His observations thus far have not yet enabled him to place the other measurements in their serial order.—*L' Union Medicale.*

